# Nerve Prostration and Other Functional Disorders of Daily Life

**Published:** 1888-12

**Authors:** 


*Nerve Prostration and other Functional Disorders of Daily Life.* By ROBSON ROOSE, M.D. London: H. K. LEWIS. 1888.

The size of this book is in inverse proportion to the gravity of the disorders of which it treats. Functional disorders have this advantage, that the patient returns again indefinitely, as the case does not come to an end. It might be entitled a text-book of medicine with the incurable diseases left out, as it extends over a wide range of subjects, from neuralgia and epilepsy to lithæmia and albuminuria. It is not quite so dry as the ordinary text-book, and might even be classed with medical light literature: it is not, however, a medical novel, inasmuch as there is little which is novel in its pages. The author has the pen of a ready writer, and has produced a fat volume, on thick paper and in good type, which gives his experience of the usual methods in vogue by which the various forms of asthenia, as they are met with in every-day work, may be treated with more or less success. We doubt not but that the preparation of the book gave its author much pleasure and profit: its perusal by others will be equally entertaining, and may be undertaken at odd moments when the newspaper fails to give either instruction or amusement. Those who have time to read much will be interested in comparing their own experience with one whose resources are great and whose observation appears to be reliable.

